# The Relationship Between Enterprise Financial Risk and R&D Investment Under the Influence of the COVID-19

**DOI:** 10.3389/fpubh.2022.910758

**Published:** 2022-08-04

**Authors:** Xinfei Li, Baodong Cheng, Yueming Li, Jingyang Duan, Yuan Tian

**Affiliations:** ^1^School of Economics and Management, Beijing Forestry University, Beijing, China; ^2^School of Business, Beijing Union University, Beijing, China

**Keywords:** financial risk, R&D investment, COVID-19, enterprise ownership, moderating variable

## Abstract

The COVID-19 pandemic has dealt a considerable blow to the development of Chinese enterprises. Therefore, exploring how to reduce the enterprise financial risk under the impact of the COVID-19 has become a current research hotspot. We select the data of 3,098 A-share companies in the quarters of 2019 and 2020, use the Z-score model to reasonably evaluate enterprise financial risk, and analyze the impact of Research and Development (R&D) investment on enterprise financial risk under the COVID-19.The results show that: ① The COVID-19 pandemic has increased the number of high-risk enterprises. ② R&D investment can effectively reduce the enterprise financial risk, and enterprises that attach importance to scientific research are relatively less affected by the COVID-19. ③ Compared with non-state-owned enterprises, R&D investment under state-owned enterprises can better help enterprises reduce financial risk. ④ When the enterprise financial risk is lower, the role of R&D investment in reducing financial risk is more significant. With the increase of financial risk, the effect of R&D investment on it is weakened. The research results are beneficial to help enterprises to correctly assess their financial risks during the COVID-19, so that enterprises can reasonably invest in research and development, and ultimately ensure the sustainable development of enterprises under the COVID-19.

## Introduction

The COVID-19 pandemic not only has a significant impact on the world economy, but also has made a great impact on the development of China's enterprises (1–3). Amid the impact of the COVID-19 pandemic, various industries have been hit hard by supply and demand due to job losses and recession fears (4, 5). Enterprises would face higher financial risks during the COVID-19. Therefore, exploring how to reduce the financial risks of enterprises under the impact of the COVID-19 has become an urgent problem to be solved. With the advent of the era of science and technology, many companies try to reduce corporate financial risks through Research and Development (R&D) investment, and exploring the relationship between the two has become a hot topic in academic circles.

The studies have paid more attention to the different effects of R&D investment on enterprise performance. However, there are few studies on the enterprise financial risks associated with R&D investment. On the one hand, some scholars believe that innovation investment can enhance future competitiveness and reduce the enterprise financial risk (6). John et al. (7) believes that R&D investment can improve the enthusiasm of enterprises to innovate, further improve the operation efficiency of enterprise capital, and help enterprises improve their ability to take financial risks. Other scholars believe that R&D investment would occupy too much capital and increase financial risks (8, 9). Innovation itself is already risky (10). R&D investment would occupy more resources, bringing many uncertainties, such as the loss of investment opportunities. This inevitably brings corresponding financial pressures (11). Huang et al. (12) adopted individual random effect model to verify the relationship between R&D investment and risk, and the research results showed that R&D investment and bankruptcy risk was significantly positive correlation. In addition, some scholars believe that the impact of R&D investment on financial risks cannot be determined (13). For example, Comin and Mulani (14) used the endogenous growth model to study the relationship between company-level volatility and R&D, and concluded that the impact of R&D investment on enterprise risk is dynamic. Hong et al. (15) found that the R&D investment of high-tech enterprises has an impact on enterprise risk, but different sources of financing have different degrees of impact.

To sum up, there are many studies on the relationship between R&D investment and enterprise financial risk, and the research conclusions are different. Although most scholars hold a very positive attitude toward the role of R&D investment, the confusion of empirical conclusions still brings great uncertainty to the practical decision-making of the government and enterprises. Therefore, based on the existing research, this paper will further empirically clarify the relationship between R&D investment and enterprise financial risk, especially whether the relationship has changed during the COVID-19. In the past, although some scholars studied R&D investment and enterprise financial risk, most of them were based on the normal period of economic development and did not involve the macro environment mutation such as the COVID-19 pandemic. To make up for the lack of existing research, we further explore the relationship between R&D investment and financial risk based on the Z scoring model. In addition, we also compare whether the impact of R&D investment on financial risk has changed before and after COVID-19.

The marginal contributions of this paper are as follows: ① R&D is an important part of enhancing technological innovation capabilities. R&D itself is accompanied by various risks. From scientific researchers, special equipment to learning and training, there are uncertainties and risks. There is a certain opportunity cost in R&D investment. The improvement of R&D investment in innovation ability can promote performance, and it would inevitably lead to financial pressure and financial risks. Therefore, research on the impact of R&D investment on financial risk has a certain reference value for companies' innovation investment decisions. This not only provides decision-making basis for managers' R&D activities, but also provides reference for policy makers. ② This research would focus on analyzing the financial risks of listed companies during the COVID-19. This paper explores the changes in financial risks caused by the COVID-19 in combination with the factors of corporate R&D investment. Under the realistic background of implementing the innovation-driven development strategy, this paper discusses the relationship between the R&D investment intensity and financial risk of enterprises in the case of a sudden change in the macro environment from a new perspective. This can provide enterprises with suggestions for mitigating financial risks in response to the impact of the COVID-19.

## Materials and Methods

### Research Assumptions

The outbreak of the COVID-19 in early 2020 led to the shutdown of many enterprises, which had a great impact on the operation of enterprises. Under the influence of COVID-19, the circulation of various production factors of enterprises is not smooth, resulting in a great impact on the profitability and asset liability structure of enterprises. This leads to the difficulty of capital turnover in the enterprise's own operation, which increases the enterprise financial risk.

Enterprises are mainly impacted by the COVID-19 in three aspects. First of all, the operating income decreased sharply. In terms of supply, except for enterprises related to prevention of COVID-19, almost all enterprises have stopped production, and it is difficult to obtain raw materials, semi-finished products and commodities required for enterprise production and operation. In terms of demand, as COVID-19 has not completely ended, the demand side has not returned to the normal level. Therefore, some small and medium-sized enterprises have plummeted orders or even no orders, facing the risk of shutdown. Secondly, the cost pressure increases. Affected by the COVID-19, the income of enterprises is significantly less, but the cost has not decreased, and some enterprises even increase the cost. Enterprises not only need to pay fixed expenses such as employee salary, house rent and bank interest. At the same time, we should also do a good job in prevention of COVID-19, such as purchasing thermometer, mask and disinfectant. Finally, the enterprise capital chain is getting tense. Under the impact of the COVID-19, the capital chain is more tense due to the sharp reduction of income, unchanged or even increased costs. Small and medium-sized enterprises, in particular, may be able to barely maintain their operations under normal circumstances. However, in case of sudden risks, the capital chain will face the risk of fracture, and the enterprise may go bankrupt directly.

**Hypothesis 1** is put forward: Under the impact of the COVID-19, the financial risk of most enterprises would increase.

According to Schumpeter's innovation theory, science and technology are the driving force of enterprise development, and the formation of enterprise core competitiveness depends on Enterprise R&D activities (16–19). Existing literature has repeatedly pointed out that R&D investment is beneficial for enterprises to reduce financial risks (7). Due to the great differences in R&D investment of various enterprises, the proportion of scientific and technological factors in output value is different. Enterprises with high R&D investment intensity are relatively less dependent on traditional production factors, so they may be less affected by the COVID-19.

**Hypothesis 2** is put forward: R&D investment would significantly reduce the enterprise financial risk regardless of COVID-19.

Corporate ownership may affect the effect of R&D investment on enterprise financial risk. For non-state-owned enterprises and state-owned enterprises, the policies of local governments are different. The development goals of enterprises are also different, which also affects the R&D investment and financial risks. R&D requires a lot of financial support, and state-owned enterprises are more likely to obtain subsidy resources than non-state-owned enterprises (20) to support innovative behavior. Moreover, the business objectives of state-owned enterprises and non-state-owned enterprises are different. Non-state-owned enterprises pursue profit maximization (21–23), and it takes time and risks to convert R&D investment into economic profits. Non-state-owned enterprises face higher risks than state-owned enterprises.

**Hypothesis 3** is proposed: the nature of the enterprise would affect relationship between R&D investment and enterprise financial risk. Compared with state-owned enterprises, R&D investment is more powerful in reducing enterprise financial risk under state-owned enterprises.

### Variable Definition

#### Explained Variable

At present, domestic and foreign scholars research financial risk models mainly include univariate models and multivariate models. The multivariate model includes Z-value model, logit model, probit model, and principal component analysis model. Compared with other models, the Z-value model is more suitable for the financial risk rating of listed companies for the following reasons: On the one hand, the logit model and probit model have a large amount of calculation. The logit model and probit model did not test the multicollinearity of the research samples, so the prediction results are questionable. In addition, both logit model and probit model take the probability equal to 0.5 as the dividing point to judge the financial situation of enterprises, which is often inconsistent with the actual situation. Finally, the Logit model and probit model require a large number of samples, and the principle of randomness should be followed when selecting research samples. Due to the limited number of listed companies in China, the sample size may not meet the requirements of these two models. Research needs, and the model does not have many requirements on the number of samples and selection principles. On the other hand, compared with the principal component analysis method, the Z model is better than the principal component analysis method in terms of the frequency of use in practice and the requirements of the preconditions. Although the financial indicators selected by the principal component analysis method are a group of comprehensive indicators that have no relationship with each other, it is difficult to determine which indicators are the main component indicators. The amount of calculation is large and difficult to understand.

The comparison between Z model and other financial crisis early warning models shows that the reason why Z model is so widely used in practice has the following advantages (24). Firstly, the calculation method is simple and operable. The data of the research samples can be obtained directly from the annual reports of listed companies. The calculation method is simple. As long as the data of relevant indicators are found and substituted into Excel software for calculation, the *Z*-value can be obtained, so as to judge the financial situation of the enterprise according to the critical value. Secondly, it has strong credibility. Since the financial situation of listed companies is audited and published by accounting firms, the data are true, and the calculated values are objective and accurate. Moreover, the selection of indicators is basically the same, and the selected indicators can comprehensively represent the financial situation of the enterprise, so the research conclusion is credible. Therefore, enterprise managers can conduct financial analysis at any time, so as to find the potential financial crisis as soon as possible and take effective measures in time to eliminate the financial crisis in the embryonic stage (25).

American scholar Altman (26) first proposed the financial risk analysis model of *Z*-score in the 1960s. He selected the same number of normal enterprises and enterprises on the verge of bankruptcy, and then collected and sorted out the financial situation of these enterprises. Finally, using the means of mathematical statistics, he determined 4–5 of more than 20 financial indicators as control variables and constructed the Z model (26). Through the comprehensive analysis of these factors, the model calculates five financial ratio indicators and obtains their *Z*-value by weighting, and obtains the corresponding *Z*-value, so as to judge whether the enterprise is facing financial crisis. At present, this analysis method is widely used in China's theoretical circles, which has high applicability and maturity. Many scholars have studied the problems of Z scoring model in enterprise operation and financial risk. Zhu et al. (27) analyzed the main risk sources faced by using Z scoring model. Srebro et al. (28) analyzed the financial risk and bankruptcy probability of listed companies. Z scoring model is often used to measure the short-term financial risk (29, 30). Z scoring model is often used to measure the short-term financial risk of enterprises. The model selects five financial index ratios and gives different weights according to the characteristics of each index. The expression of the model is as follows.


(1) $$\begin{array}{rcl} Z & = & 1.2X_{1}+1.4X_{2}+3.3X_{3}+0.6X_{4}+0.999X_{5} \end{array}$$

(2) $$\begin{array}{l} {\text{X}}_{1}\text{= Working Capital }(\text{WC})\text{/Total Assets }(\text{TA})\\ \text{ = }(\text{Current Assets }-\text{ Current Liabilities})\text{/Total Assets} \end{array}$$

(3) $$\begin{array}{l} {\text{X}}_{2}\text{= Retained Earnings }(\text{RE})\text{/Total Assets }(\text{TA})\\ \text{ = }(\text{Undistributed Profit +Surplus Reserve})\text{/Total Assets} \end{array}$$

(4) $$\begin{array}{l} {\text{X}}_{3}\text{= Earnings Before Interest and Tax }(\text{EBIT})\text{/Total Assets }(\text{TA})\\ \text{ = }(\text{Profit before Tax + Interest Expense})\text{/Total Assets} \end{array}$$

(5) $$\begin{array}{l} {\text{X}}_{4}\text{= Market Value of Equity }(\text{MVE})\text{/Book Value of Total Debt}\\ \;(\text{BVD})\text{ = Total Market Value/Total Liabilities} \end{array}$$

(6) $$\begin{array}{l} {\text{X}}_{5}\text{= Sales revenue }(\text{S})\text{/Total Assets }(\text{TA})\\ \text{ = Main Business Revenue/Total Assets} \end{array}$$

### Other Variables

#### Explanatory Variable

R&D expense ratio (RD), that is, the proportion of R&D expenses in the company's operating revenue. We use the index as an explanatory variable to describe the importance of enterprises to science and technology investment.

#### Moderating Variable

Moderating variable: in order to explore the impact of R&D investment intensity on enterprise performance under different internal governance, this paper introduces the corporate nature moderating variable (STATE). If the actual controller of the listed company is state-owned shares, state shares and state-owned legal person shares, the value of state is 1, otherwise it is 0.

#### Control Variables

Based on previous literature experience, this paper sets the Return on Equity (ROE), Return on Assets (ROA) and Return on Total Assets Ratio (RAR) as control variables, and sets industry dummy variables according to the China Securities Regulatory Commission (CSRC) to control each sub industry of the industry of the selected sample.

### Construction of Regression Equation

Taking Z index as dependent variable and RD, ROE, ROA, RAR and Industry index as independent variables, the regression equation is established as follows. ∑*IndustryDummy* and ∑*TimeDummy* mean that the models control the industry effect and time effect. ε_*i, t*_ is the random error term. The model 1 is as follows


(7) $$\begin{array}{l} \text{lnZ = }\alpha_{\text{0}}\text{+}\alpha_{\text{1}}\text{RD + }\alpha_{\text{2}}\text{ROE + }\alpha_{\text{3}}\text{ROA + }\alpha_{\text{4}}\text{RAR}\\ \text{ +}\sum IndustryDummy\text{ +}\sum TimeDummy\text{ +}\varepsilon_{i,t} \end{array}$$

In order to verify whether enterprise nature can regulate the effect of RD on enterprise performance, this paper adds the regulating variable enterprise nature (STATE) on the basis of model (1). The model 2 is as follows


(8) $$\begin{array}{l} \text{lnZ = }\alpha_{\text{0}}\text{+ }\alpha_{\text{1}}\text{RD + }\alpha_{\text{2}}\text{STATE + }\alpha_{\text{3}}\text{RD }*\text{ STATE + }\alpha_{\text{4}}\text{ ROE}\\ \text{ +}\alpha_{\text{5}}\;ROA\text{ + }\alpha_{\text{6}}\;RAR\text{+}\sum IndustryDummy\\ \text{ +}\sum TimeDummy\text{ + }\varepsilon_{i,t} \end{array}$$

### Data Selection

This study selects the data of listed enterprises in the industry classification of the China Securities Regulatory Commission (CSRC), excludes the samples with incomplete B shares, ST shares and financial data, and obtains a sample of 3,098 companies. On this basis, obtain their financial data during the 2019 quarterly report (before the COVID-19) and 2020 quarterly report, and calculate the *Z*-value. The data comes from Guotai'an database. In this paper, the data of the four quarters in 2019 are set as group A (Before the spread of COVID-19) and the data of the four quarters in 2020 are set as group B (after the spread of COVID-19).

## Results

### Descriptive Statistics

**Figure 1:**
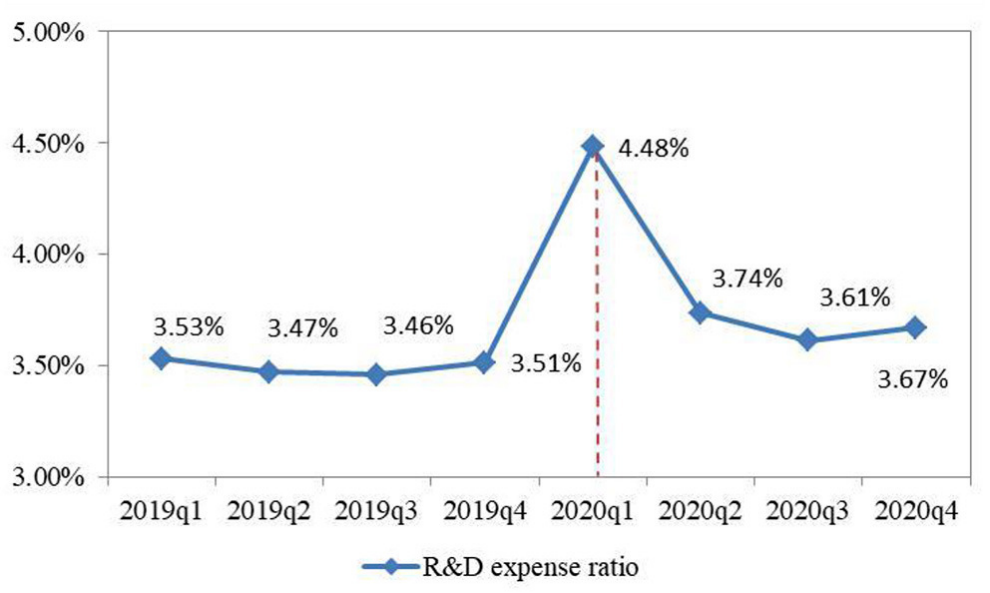
R&D expense rate of listed companies from 2019 to 2020.

This paper describes the R&D expense ratio (RD) of 3098 listed companies in the four quarters from 2019 to 2020, as shown in Figure 1. As can be seen from Figure 1, the R&D expense ratio (RD) of listed companies increased rapidly in the first quarter of 2020, becoming the highest point in four quarters. This is probably caused by the COVID-19. Under the influence of the COVID-19, on the one hand, listed enterprises have strengthened R&D investment, so that enterprises can withstand various risks brought by sudden changes in the macro environment. On the other hand, the operating income of enterprises has decreased significantly in a short time, because the R&D expense ratio (RD) has increased significantly in the first quarter of 2020.

Enterprises are divided into three categories according to *Z*-value. This paper refers to the research of Pei et al. (31) and Hong et al. (14), *Z*-value ≥3 is defined as a low-risk group with good financial condition; When the *Z*-value is in the range of (1.8, 3), it is defined as a medium risk group, which has certain financial hidden dangers. When Z is ≤1.8, it is defined as a high-risk group, that is, the financial risk is great. In this paper, the data of the four quarters in 2019 are set as group A, that is, before the outbreak, and the data of the four quarters in 2020 are set as group B, that is, after the outbreak, descriptive statistics are carried out for groups A and B based on the *Z*-value results.

As shown in Table 1, among the 3,098 groups of comparison samples before and after the COVID-19.The average value of high-risk samples in the four quarters of 2020 increased by 147 compared with that before the COVID-19, medium-risk samples decreased by 87, and low-risk samples decreased by 60. This shows that under the impact of the COVID-19, the financial risk of most enterprises will increase to a certain extent. **Hypothesis 1** can be verified.

**Table 1:** Relationship between *Z*-value and financial risk.

***Z*-value**	**Financial risk of industry**
Z ≥ 3	In good financial condition, in the low-risk group
1.8 < Z <3	There are hidden dangers and they are in the medium financial risk group
Z ≤ 1.8	The financial risk is very high and is in the high-risk group

### Empirical Analysis

In order to make it easier to compare the impact of R&D investment on corporate financial risks before and after the outbreak, in addition to constructing the full sample model for 2019–2020, this paper also separately analyzes the 2019 sample A and 2020 sample for regression analysis. The regression results are shown in Table 2. In addition, the model was tested for heteroscedasticity and multicollinearity. Taking Model 1 as an example, the White test was performed on the model. The test result showed that *p* > 0.1, that is, the null hypothesis could not be rejected, and the model had no heteroscedasticity. Similarly, this paper tested the multicollinearity of the model, and the variance inflation factor (VIF) value was only 6.39, which was much lower than 10, so the multicollinearity could basically be ignored.

**Table 2:** Regression results of R&D investment.

**Variables**	**Full sample**		**A sample**		**B sample**	
	**Model 1**	**Model 2**	**Model 3**	**Model 4**	**Model 5**	**Model 6**
RD	2.955^***^	2.828^***^	3.245^***^	3.024^***^	2.771^***^	2.694^***^
	(0.0962)	(0.118)	(0.142)	(0.167)	(0.131)	(0.165)
STATE		−0.0377^***^		−0.0473^***^		−0.0316^**^
		(0.0103)		(0.0140)		(0.0150)
RD*STATE		0.282^*^		0.589^**^		0.149
		(0.171)		(0.256)		(0.233)
ROA	21.01^***^	21.11^***^	23.00^***^	23.08^***^	20.00^***^	20.10^***^
	(0.485)	(0.485)	(0.672)	(0.672)	(0.722)	(0.723)
RAR	−0.0997^***^	−0.0993^***^	−0.0923^***^	−0.0918^***^	−0.149^***^	−0.149^***^
	(0.0116)	(0.0116)	(0.0117)	(0.0117)	(0.0380)	(0.0380)
ROE	−13.78^***^	−13.87^***^	−16.33^***^	−16.40^***^	−12.15^***^	−12.25^***^
	(0.453)	(0.454)	(0.637)	(0.637)	(0.653)	(0.655)
Industry	Control	Control	Control	Control	Control	Control
Time	Control	Control	Control	Control	Control	Control
Constant	1.074^***^	1.090^***^	1.088^***^	1.108^***^	1.036^***^	1.051^***^
	(0.0388)	(0.039)	(0.0522)	(0.0525)	(0.0566)	(0.0570)
Observations	24,784	24784	12392	12,392	12,392	12,392
R-squared	0.312	0.312	0.321	0.322	0.309	0.310

^*^, ^**^, and ^***^ respectively represent that the estimated coefficient is significant at the confidence level of 10, 5, and 1%, and the standard error of the coefficient is marked in parentheses.

From model 1 and model 2, it can be seen that the increase in R&D investment during 2019–2020 will significantly increase the *Z*-value. As mentioned above, the higher the *Z*-value, the lower the financial risk. It means that the increase in R&D investment can significantly reduce the financial risk of the enterprise. Based on this, we further compared the variables of the impact of R&D investment on the *Z*-value in Group A in 2019 and Group B in 2020 before the COVID-19 pandemic. It can be seen from model 4 and model 6 that although R&D investment can significantly reduce financial risk in 2019 and 2020, the impact coefficient has changed. After the outbreak of the COVID-19, although R&D investment still has a significant positive impact on the *Z*-value, the impact coefficient has dropped significantly compared to 2019 before the outbreak. The research results show that R&D investment can significantly reduce financial risks before and after the COVID-19, and the impact of R&D investment before the COVID-19 is greater. That is, **Hypothesis 2** is verified.

In addition, the interaction term (RD^*^STATE) of the enterprise nature has a significant negative impact on Z in full sample and A sample. In sample B, although the impact of interaction term (RD^*^STATE) on Z is not significant, it is still positive. This result shows two points. First, if the enterprise is state-owned, it would increase the positive impact of R&D investment on the *Z*-value. The higher the *Z*-value, the lower the financial risk. In other words, compared to non-state-owned enterprises, under state-owned enterprises, R&D investment can better help enterprises mitigate financial risks. **Hypothesis 3** is verified. Second, in sample B after the outbreak of COVID-19, although the interaction item (RD^*^STATE) has a positive impact on Z, it is not significant. This shows that after the COVID-19, the regulatory effect of enterprise nature on R&D investment in financial risk becomes smaller.

### Robustness Check

#### Explanatory Variable Lag

As shown in Table 3, in order to test the documentation of the model, the explanatory variable RD is lagged by one period. The research results show that the R&D investment lagging one phase can significantly increase the *Z*-value, both before and during the COVID-19. The effects of the control variables are also basically the same as in the original model, which speaks to the credibility of the model results.

**Table 3:** Regression results of R&D investment lagging behind phase I.

**Variables**	**Full sample**		**A sample**		**B sample**	
	**Model 7**	**Model 8**	**Model 9**	**Model 10**	**Model 11**	**Model 12**
RD-1	3.165^***^	2.952^***^	2.917^***^	2.610^***^	3.301^***^	3.119^***^
	(0.0998)	(0.112)	(0.152)	(0.175)	(0.131)	(0.146)
STATE		−0.0496^***^		−0.0611^***^		−0.0447^***^
		(0.0105)		(0.0158)		(0.0142)
RD*STATE		0.648^***^		0.986^***^		0.542^***^
		(0.165)		(0.290)		(0.204)
ROA	20.74^***^	20.83^***^	23.42^***^	23.47^***^	19.49^***^	19.60^***^
	(0.512)	(0.513)	(0.727)	(0.728)	(0.717)	(0.718)
RAR	−0.311^***^	−0.311^***^	−0.484^***^	−0.482^***^	−0.146^***^	−0.146^***^
	(0.0261)	(0.0261)	(0.0354)	(0.0354)	(0.0377)	(0.0377)
ROE	−13.18^***^	−13.25^***^	−16.04^***^	−16.08^***^	−11.73^***^	−11.81^***^
	(0.471)	(0.471)	(0.681)	(0.681)	(0.649)	(0.650)
Industry	Control	Control	Control	Control	Control	Control
Time	Control	Control	Control	Control	Control	Control
Constant	1.031^***^	1.051^***^	1.025^***^	1.048^***^	1.031^***^	1.050^***^
	(0.0412)	(0.0415)	(0.0586)	(0.0589)	(0.0562)	(0.0565)
Observations	21,686	21686	9294	9,294	12,392	9,294
R-squared	0.331	0.332	0.361	0.362	0.319	0.320

^*^, ^**^, and ^***^ respectively represent that the estimated coefficient is significant at the confidence level of 10, 5, and 1%, and the standard error of the coefficient is marked in parentheses.

#### Add Dummy Variable

Table 4 further examines whether the ability of R&D investment to reduce financial risks is affected after COVID-19 pandemic. We put before and after the COVID-19 pandemic as dummy variables (COVID) into the model. The COVID-19 pandemic started in January 2020, so the dummy variable COVID is 0 in 2019 and 1 in 2020. The results are shown in Table 4, where model 13 is the original model that only considers R&D investment, model 14 considers both R&D investment and COVID-19 pandemic. The model 15 adds the interaction term (RD^*^COVID) about R&D investment and COVID-19 pandemic.

**Table 4:** Whether the outbreak of the COVID-19 affects the impact of R&D investment on the financial risk of enterprises.

**Variables**	**Full sample**		
	**Model 13**	**Model 14**	**Model 15**
RD	2.955^***^	2.955^***^	3.277^***^
	(0.0962)	(0.0962)	(0.139)
COVID		−0.0365^***^	−0.0168^*^
		(0.00805)	(0.0101)
RD*COVID			−0.540^***^
			(0.169)
ROA	21.01^***^	21.01^***^	20.96^***^
	(0.485)	(0.485)	(0.485)
RAR	−0.0997^***^	−0.0997^***^	−0.0994^***^
	(0.0116)	(0.0116)	(0.0116)
ROE	−13.78^***^	−13.78^***^	−13.74^***^
	(0.453)	(0.453)	(0.453)
Industry	Control	Control	Control
Time	Control	Control	Control
Constant	1.074^***^	1.074^***^	1.063^***^
	(0.0388)	(0.0388)	(0.0389)
Observations	24,784	24,784	24,784
R-squared	0.312	0.312	0.312

^*^, ^**^, and ^***^ respectively represent that the estimated coefficient is significant at the confidence level of 10, 5, and 1%, and the standard error of the coefficient is marked in parentheses.

From model 13 and model 14, it can be seen that R&D investment has a significant positive effect on Z, and the COVID-19 pandemic has a significant negative effect on Z. The higher the *Z*-value, the lower the financial risk. Therefore, R&D investment can significantly reduce the enterprise financial risk, and the COVID-19 pandemic would significantly increase the financial risk. The model 15 further verifies whether the COVID-19 can affect the relationship between R&D investment and Z. From the results of interaction items (RD^*^COVID), the COVID-19 reduces the positive impact of R&D investment on *Z*-value. That is, although R&D investment is still possible to reduce financial risks, but the ability to influence is significantly lower than before COVID-19 pandemic.

## Discussion

Based on the background of COVID-19 outbreak, the paper uses the Z-score model to reasonably evaluate enterprise financial risk. The results show that COVID-19 pandemic has increased the number of high-risk enterprises in China, but R&D investment can significantly reduce enterprise financial risk. In addition, we further explore whether the impact of R&D investment on financial risk is different in companies with different risk levels based on the above analysis results. We conduct group regression according to the enterprise risk classification results above, and the results are shown in Table 5.

**Table 5:** The impact of R&D investment on corporate financial risks under different risk companies.

**Variables**	**Low risk**	**Medium risk**	**High risk**
	**Model 16**	**Model 17**	**Model 18**
RD	1.505^***^	0.505^***^	0.0561
	(0.0735)	(0.0552)	(0.193)
ROA	18.35^***^	4.064^***^	4.392^***^
	(0.568)	(0.298)	(0.729)
RAR	−3.988^***^	−0.998^***^	−0.0191^*^
	(0.125)	(0.0922)	(0.00977)
ROE	−8.631^***^	−1.045^***^	−2.615^***^
	(0.458)	(0.234)	(0.731)
Industry	Control	Control	Control
Time	Control	Control	Control
Constant	1.469^***^	0.857^***^	0.0398
	(0.0323)	(0.0171)	(0.0735)
Observations	12,750	6,399	5,635
R-squared	0.184	0.091	0.061

^*^, ^**^, and ^***^ respectively represent that the estimated coefficient is significant at the confidence level of 10, 5, and 1% and the standard error of the coefficient is marked in parentheses.

According to the results, it can be seen that R&D investment can significantly increase the *Z*-value in enterprises with low and medium financial risks. The higher the *Z*-value, the lower the financial risk. That is, in low-risk and medium-risk enterprises, R&D investment can significantly reduce the enterprise financial risk. It is worth noting that with the increase of enterprise risk, the effect of R&D investment on enterprise financial risk is gradually weakened. In high-risk enterprises, although R&D investment still has a positive effect on Z, it is not significant. The reason why this happens is that, first, the increase in R&D investment requires financial support, and second, it takes a certain amount of time for R&D investment to be transformed into results before it can finally have an economic effect. When companies are already facing huge financial risks, increasing R&D investment is obviously useless to financial risks and even makes economic pressure even greater.

## Research Conclusion

We use the Z-score model to reasonably evaluate enterprise financial risk, and analyze the impact of Research and Development (R&D) investment on enterprise financial risk under the COVID-19.The results show that: ① The COVID-19 pandemic has increased the number of high-risk enterprises. ② R&D investment can effectively reduce the enterprise financial risk, and enterprises that attach importance to scientific research are relatively less affected by the COVID-19. ③ Compared with non-state-owned enterprises, R&D investment under state-owned enterprises can better help enterprises reduce financial risk. ④ When the enterprise financial risk is lower, the role of R&D investment in reducing financial risk is more significant. With the increase of financial risk, the effect of R&D investment on it is weakened.

The research results show that although the positive effect of R&D investment on reducing financial risks would be reduced during COVID-19, it can still help enterprises effectively reduce financial risks to a certain extent. That is, enterprises that attach importance to scientific research are relatively less affected by the COVID-19. We put forward suggestions from both the enterprise and the government.

On the one hand, enterprises should increase investment in research and development and improve the structure of scientific research investment to resist changes in financial risks caused by emergencies such as COVID-19. However, R&D investment needs to be combined with its own development. When the enterprise itself is already in a state of high risk, R&D investment cannot effectively help enterprises reduce risks. Therefore, enterprises should improve production technology, adjust industrial structure, and effectively reduce risks based on economic conditions and market needs.

On the other hand, the government should formulate relevant policies to help companies invest in R&D, such as financial support and the establishment of incentive mechanisms. Especially non-state-owned enterprises, non-state-owned enterprises pay more attention to short-term economic benefits. R&D investment not only requires a lot of capital, but also takes a certain amount of time to convert R&D investment into production technology. Many non-state-owned enterprises are not active in R&D investment. Therefore, the government should strengthen the supervision of non-state-owned enterprises, help non-state-owned enterprises to innovate and transform, and take the road of sustainable development.

Due to the limitation of our ability, further research is needed to solve some unsolved problems and imperfect parts. First, the Z model is also insufficient to assess corporate financial risk. In addition, due to the outbreak in January 2020, the sample time was short.

## Data Availability Statement

Publicly available datasets were analyzed in this study. This data can be found here: www.gtarsc.com/.

## Author Contributions

XL and YT conceived and designed the study. XL, YT, and BC designed the model and contributed to the writing of the manuscript. JD and YL collected the parameters. XL, YT, and YL did the data analyses. All authors interpreted the results and approved the final version for publication.

## Funding

This research was funded by Beijing Social Science Foundation (21LLGLC038), Humanities and Social Science Foundation of Ministry of Education of China (21YJC630127), Social Science Program of Beijing Municipal Education Commission (SM202011417010), and National Natural Science Foundation of China (Grant Nos. 72073012 and 71873016).

## Conflict of Interest

The authors declare that the research was conducted in the absence of any commercial or financial relationships that could be construed as a potential conflict of interest.

## Publisher's Note

All claims expressed in this article are solely those of the authors and do not necessarily represent those of their affiliated organizations, or those of the publisher, the editors and the reviewers. Any product that may be evaluated in this article, or claim that may be made by its manufacturer, is not guaranteed or endorsed by the publisher.
